# The use of a low cost 3D scanning and printing tool in the manufacture of custom-made foot orthoses: a preliminary study

**DOI:** 10.1186/1756-0500-7-443

**Published:** 2014-07-10

**Authors:** Colin E Dombroski, Megan ER Balsdon, Adam Froats

**Affiliations:** 1 Faculty of Health Science, Department of Physical Therapy, Western University, London, ON N6A 5B9, Canada; 2 SoleScience Inc., Fowler Kennedy Sports Medicine Clinic, 3M Building, Western University, London, ON N6A 5B9, Canada

**Keywords:** Custom-made foot orthoses, 3D printing, Microsoft Kinect, plaster casting

## Abstract

**Background:**

Custom foot orthoses are currently recognized as the gold standard for treatment of foot and lower limb pathology. While foam and plaster casting methods are most widely used in clinical practice, technology has emerged, permitting the use of 3D scanning, computer aided design (CAD) and computer aided manufacturing (CAM) for fabrication of foot molds and custom foot orthotic components. Adoption of 3D printing, as a form of CAM, requires further investigation for use as a clinical tool.

This study provides a preliminary description of a new method to manufacture foot orthoses using a novel 3D scanner and printer and compare gait kinematic outputs from shod and traditional plaster casted orthotics.

**Findings:**

One participant (male, 25 years) was included with no lower extremity injuries. Foot molds were created from both plaster casting and 3D scanning/printing methods. Custom foot orthoses were then fabricated from each mold. Lower body plug-in-gait with the Oxford Foot Model on the right foot was collected for both orthotic and control (shod) conditions. The medial longitudinal arch was measured using arch height index (AHI) where a decrease in AHI represented a drop in arch height. The lowest AHI was 21.2 mm in the running shoes, followed by 21.4 mm wearing the orthoses made using 3D scanning and printing, with the highest AHI of 22.0 mm while the participant wore the plaster casted orthoses.

**Conclusion:**

This preliminary study demonstrated a small increase in AHI with the 3D printing orthotic compared to the shod condition. A larger sample size may demonstrate significant patterns for the tested conditions.

## Findings

Custom foot orthoses have been linked to reduced risk and recurrence of injury through individualization of cushioning and support features [1]. Applications include, but are not limited to: pain relief, increased heel cushion, correction of flexible deformity, increased foot stability and/or prevention of skin breakdowns, such as ulceration [2]. Custom foot orthoses are currently recognized as the gold standard of treatment for foot and lower limb pathology, offering the benefits of individualized prescription in comparison to over-the-counter “best fit” devices [3].

Current best practice methods used to obtain a three-dimensional (3D) impression of a patients’ foot for use in orthoses manufacturing are plaster and foam box casting. Each of these casting methods is subject to practitioner error and may result in excess waste [4]. Additionally, when compared to the relative ease of foam box casting, the plaster bandage method can be time consuming and cumbersome [5].

In recent years, technology has emerged permitting the use of 3D foot scanning, computer aided design (CAD), and computer aided manufacturing (CAM) in the fabrication of foot molds and custom foot orthosis components [6]. Three-dimensional (3D) printing, one of the most recent forms of CAM, has proven efficacy in the fabrication of ankle foot orthoses (AFO) with reports of excellent dimensional accuracy, good manufacturing precision, and performance that is at least equivalent to hand-crafted AFO’s [7-9]. Recent studies report positive subjective comfort ratings and similar biomechanical gait parameters with the use of orthoses manufactured using 3D printing in comparison to orthoses fabricated using traditional means [1,6,10].

Mass adoption of 3D printing for foot orthoses fabrication will require proven performance, value for money, and a good service model [10]. Currently, many 3D scanning tools, CAD programs and laser sintering machines are inaccessible to many professionals due to the high cost of acquisition. A model using an easily accessible and affordable system, therefore, would have tremendous clinical applicability if proven valid.

### Research purpose

The goal of this preliminary study was to investigate the feasibility of a low cost 3D scanning and printing arrangement that would be fairly accessible to all foot care practitioners. The kinematics of the foot while walking with custom foot orthoses made from the casting method was compared a shod condition as well as to orthotics made with a novel CAM method consisting of the Microsoft Kinect (Microsoft Corporation, Redmond, WA), open source software (MeshLab, a 3D-CoForm project; SourceForge.net) and a desktop 3D printer (Makerbot®, Makerbot Industries, Brooklyn, NY).

## Methods

One male participant, age 25, was included, with no lower extremity injuries or abnormalities. The participant provided informed consent as required by the Western University Research Ethics Board. The participant's right foot and ankle were held in the subtalar neutral position, defined by Root et al. [11] while the Microsoft Kinect scanned the participant’s foot to a stereo lithography file (.STL) on its internal hard drive. The 3D scan was then filled, smoothed and edited using open source software before being sent to the 3D printer, where a positive foot mold was printed in acrylonitrile butadiene styrene (ABS) filament, layered at 2 microns. A second foot impression was acquired using the traditional plaster casting method described by Root (1971) where the participant, prone, was held in a non-weightbearing position as dampened plaster strips dried and formed a negative positive foot impression. A positive foot mold was then created by filling the plaster impression with gauging plaster.

**Figure 1:**
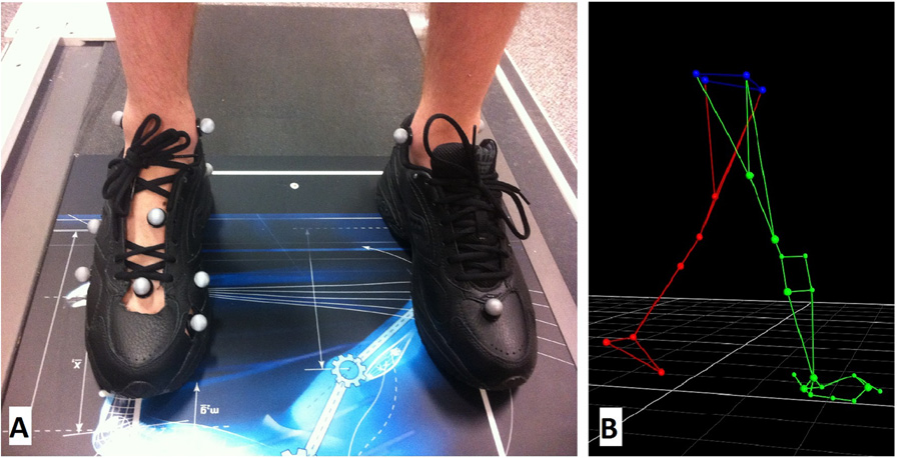
**The Oxford Foot Model marker configuration for static stance and dynamic walking.** Image **(A)** is a photograph of the static stance position with 13 foot and ankle markers, where image **(B)** shows a still image of a walking trial in Vicon Nexus with lower body plug-in-gait and the Oxford Foot Model showing 10 markers on the right foot and ankle complex.

Custom-made orthoses were fabricated from both foot molds. A Vicon motion capture system (Vicon Motion Systems Ltd, Oxford, UK) with 6 Bonita cameras was used along with twenty-eight (28) passive reflective markers, placed on the participant to define the lower body for standard Nexus plug-in gait and the Oxford Foot Model (OFM) [12] for the right foot. A static standing trial was first collected to determine joint centers (Figure 1A). The single participant completed a 30-second warm up walk at 1.3 m/s, followed by three trials of approximately ten gait cycles at 1.3 m/s collected for each condition: control (running shoe), orthotic made from 3D printed casts and orthotic made from the plaster casting method (Figure 1B). The running shoe used throughout data collection was the Saucony Grid Omni Walker, model #5260. To allow for marker placement, windows were cut into the shoe following the previously reported methods of Shultz and Jenkyn so as to not disrupt shoe integrity during gait testing [13].

An Arch Height Index (AHI) measure using the OFM was compared between the averages of each condition in Vicon Polygon. The AHI is a measure of the rigidity of the forefoot segment, used as a quality measure to check the accuracy of the model's assumption of forefoot rigidity. AHI is also an estimate of arch height, that is, the normal distance of the plane of the forefoot from the first metatarsal base.

## Results

**Figure 2:**
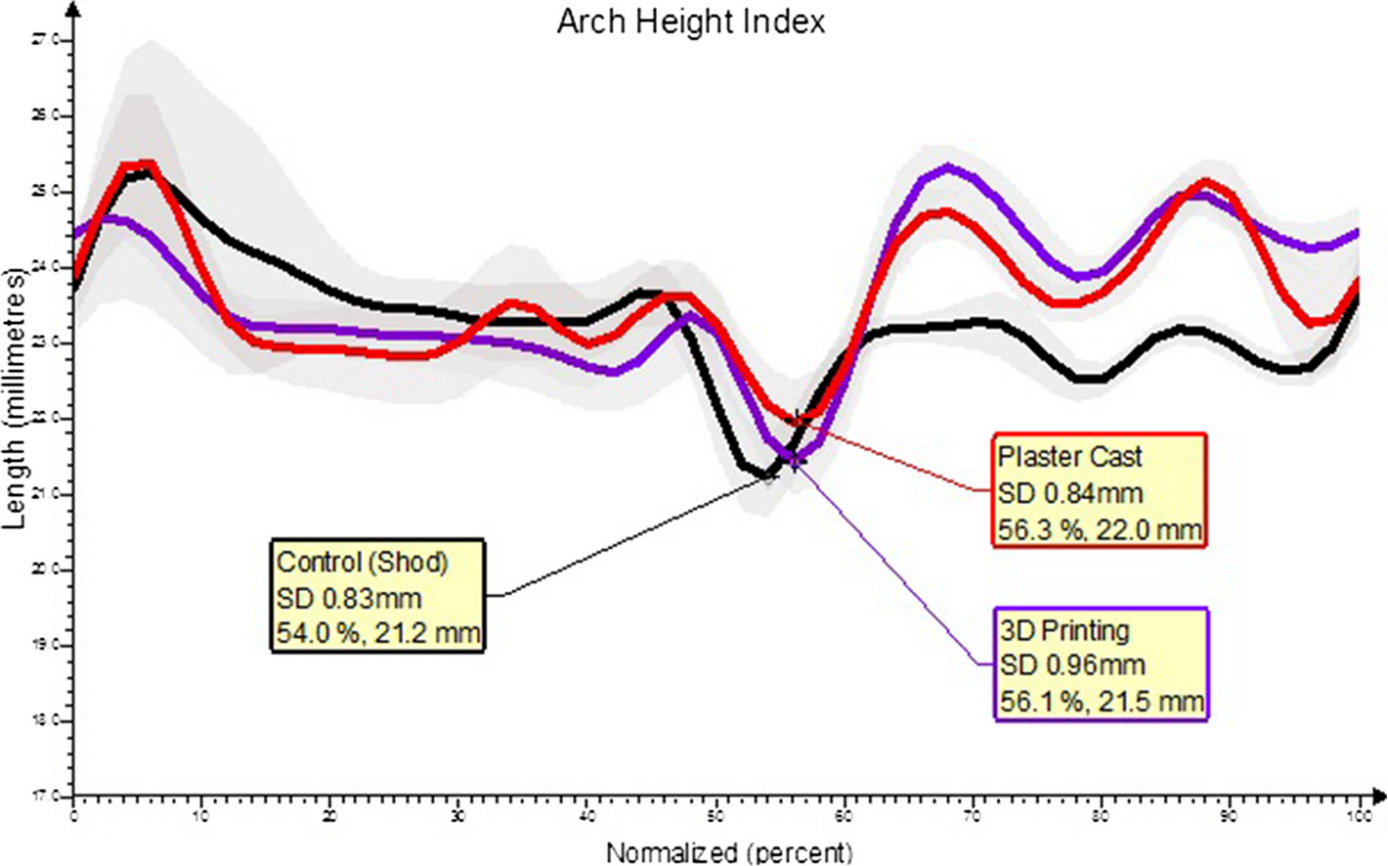
**The Arch Height Index of the right foot measured using the Oxford Foot Model.** Mean values, in millimeters (mm), from 30 gait cycles are illustrated for each trial, with (+/-) 1 standard deviation (SD) represented by the shaded area. Three conditions were compared: Control (shod), 3D Printing Orthotic and Plaster Casting Orthotic.

Three trials of ten gait cycles were averaged for each condition, totaling approximately 30 gait cycles. During midstance, the plaster casted orthotic provided the most control over movement of the medial longitudinal arch (Figure 2). This movement was measured by arch height index (AHI) where a decrease in AHI, measured in millimeters, represented a drop in medial longitudinal arch height. The lowest AHI was 21.2 mm (SD 0.83 mm) in the running shoes with standard sock liners, followed by 21.4 mm (SD 0.96 mm) with the 3D printing orthotic, and finally the highest AHI of 22.0 mm (SD 0.84 mm) while the participant wore the plaster casted orthotics. One standard deviation within the mean of each trial is shown in the shaded areas (Figure 2).

## Discussion

The orthotic made from the 3D printing method resulted in a higher AHI than the shod condition, indicating that the orthotic restricted motion of the medial longitudinal arch during midstance. The differences between the three conditions were minimal (within 0.8 mm) and variability was similar with standard deviations within 0.13 mm; however, statistical analysis was not performed since the findings are based on only one participant. The main limitation of this study is the sample size of only one, providing only a preliminary description of this developing model. Testing additional participants may reveal a more significant trend in the outcome measures using this highly affordable and accessible 3D scanning and printing method.

Using the Microsoft Kinect system has not been documented for this exact application; however, this scanning method has been validated in reproducing depth data for indoor mapping applications [14], kinematic strategies of postural control [15] and spatiotemporal gait variables [16]. Other optical scanners have been validated and are currently used in a clinical setting; therefore, a separate study should be completed to validate this particular scanning device for future use in fabrication of foot molds with 3D printing methods.

## Conclusion

These findings provide evidence that this method of fabricating foot orthoses produces an in-shoe device that results in a similar AHI measure in comparison to a traditionally made orthotic. With an increase in sample size of healthy individuals, a more in depth investigation could compare orthotics made from this method to both foam and plaster casted orthotics, and analyze multiple kinematic differences between the devices. If proven successful in the future, this model could be a low cost method of custom foot orthotic manufacturing, thus controlling the ultimate cost to the clinician and end user.

## Consent

Written informed consent was obtained from the participant for publication of this report and any accompanying images.

## Abbreviations

3D: Three-dimensional; ABS: Acrylonitrile butadiene styrene; AFO: Ankle foot orthosis; AHI: Arch height index; CAD: Computer aided design; CAM: Computer aided manufacturing; OFM: Oxford Foot Model; STL: Stereo Lithography.

## Competing interests

CD is the owner of SoleScience Inc. and the pedorthic clinic where this self-funded research took place. MB is a research assistant at SoleScience Inc. and AF is a pedorthist at SoleScience Inc. as well as a graduate student at Western University.

## Authors’ contributions

All three authors contributed to the collection of data. CD was the originator of the study concept and design, the casting and production of both orthotics as well as the study methodology and manuscript draft. MB was involved in tracking the gait data and the results of the study. AF was involved in drafting the manuscript and researching the background information. All authors read and approved the final manuscript.

## Authors’ information

CD is an Adjunct Research Professor at Western University, as well as a Canadian Certified Pedorthist within the Fowler Kennedy Clinic on the campus of Western University. CD is a member of the College of Pedorthics and the Pedorthic Association of Canada.
